# Dr. Waller on Compression of the Carotids

**Published:** 1848-10-01

**Authors:** Augustus Waller


### ON THE COMPRESSION OF THE CAROTIDS—ITS EFFECTS ON HEADACHE, EPILEPSY, HYSTERIA, &c.

BY AUGUSTUS WALLER, M.D.

The carotid arteries are seated so superficially, their pulsations so manifest, that it is not surprising that their compression, so as to interrupt the flow of "animal spirits," in the opinion of the ancients, and the course of the arterial blood according to our more correct notions of physiology, should have been tried even at the earliest period of medical science. Serapion mentions the compression of these canals as a means of curing pains in the head, but from his time I am not aware of any mention of this fact until a very recent period, when the subject was again taken up by the late Dr. Parry, of Bath.

My attention was first directed to this subject by the relief it gave me when suffering from obstinate pains of the head. I was unaware that any researches had been made on this point. On the present occasion, it is my intention to give the results I have obtained by using this process in various cases, and at the same time endeavour to show the importance of it in elucidating, in an experimental way, some of the physiological and pathological actions of the cerebral hemispheres.

It is unnecessary to enter into details respecting the distribution of the carotid and vertebral arteries, which supply the entire head with blood. It is requisite, however, to bear in mind the following particulars. The two vertebral arteries, either before or after their inosculation in one trunk, supply with blood the upper portion of the spinal marrow, the medulla oblongata, the pons Varolii, the cerebellum, and the tubercula quadragemina. The anterior and middle lobes of the brain, the corpora striata and optic thalami are supplied by the carotid arteries. The posterior lobes of the brain are provided with blood from the posterior cerebral and the posterior communicating arteries. The first arising from the vertebrals, the latter from the internal carotids. At the base of the brain, communicating branches exist between the four supplying vessels, which renders it possible for one of these arteries to keep up the circulation of the brain even where the rest are obliterated. This has been effected on man and animals by disease, or by surgical operation, and in such cases the communicating branches are found to be increased in proportion to the current required to nourish the parts beyond. Sir A. Cooper's experiments on this point are well known, therefore I shall not dwell upon them, but consider it sufficient to mention that he obtained the following results. Ligature of the carotids had no serious effect; he merely observed acceleration of the movements of respiration and of the heart. Ligature of one vertebral of a dog caused dyspnœa, which increased after the second ligature was made; to this there succeeded quickened action of the chest and heart, and weakness of the four extremities. Ligature of the four vessels was constantly fatal to rabbits, but not always so to dogs. After ligature of the carotids, compression of the vertebral arteries caused respiration to cease, and convulsive struggles to ensue. Ligature of the carotid, when performed by man, has been attended with various results, (see "Carotide Dict. des Sciences Medicales,) which some have endeavoured to explain by the fact, that the communicating branches at the base of the brain are in some cases voluminous, and in others but little developed. I have tried to ascertain how far compression of a main trunk may be compensated by a flow from the inosculating branches. By a simple experiment we may determine the manner in which the blood circulates in the brain when a carotid is compressed, and are able to decide whether the arteries are distributed in a normal manner. We are aware that the internal carotid gives off in the interior of the cranium a branch called the ophthalmic artery, of which a considerable branch, after passing through a separate foramen of the orbit, takes the name of the supra-orbital artery, and ramifies over the forehead. The pulsations of this last are very perceptible in ordinary cases; and a little consideration will show, that they may serve as a faithful index of the state of the other branches of the carotid, which are concealed from direct observation. In the cases I have examined for this purpose, compression of one carotid arrested all pulsation of the corresponding supra-orbital artery, and diminished slightly the strength of the other on the opposite side. The pulsations of both were entirely obliterated when the two carotids were compressed. We may therefore conclude that the circulation in the same hemisphere was acted upon in like manner.* I am convinced that examination of the circulation of the brain, in the mode above described, might be valuable in certain diseased conditions of that organ. After ligature of a carotid, for instance, it would be important to ascertain when the pulsations re-appear in the supra-orbital artery.

As the jugular vein accompanies each of these vessels, it is advisable not to establish the compression over too large a surface, as the return of the venous blood might be prevented. My usual mode of proceeding is to apply the extremities of the two forefingers, or that of the thumb, over the pulsating vessel. It is always advisable to try the effects of compression on both arteries. It is easy to ascertain if it is effectually applied, by placing the disengaged hand on either the facial or supra-orbital branches.†

*External pains of the head.*—These pains, which are seated generally in the integuments, are frequently relieved by arresting the flow of blood in the carotids. To choose an instance of the most simple description, we may mention the following :—In a case of imperfect amaurosis, a blister with Gondret's ointment; which takes effect in a few minutes, was placed on the sinciput, and caused a severe pain of a throbbing nature, isochronous with the beating of the arteries, and a sensation of excessive weight in the head. Compression of the carotid on either side, immediately caused considerable diminution of the pain, and slightly relieved the oppression of the head. As a general rule, external pains of the head, depending on an afflux of blood, are much alleviated by arresting the arterial current; but those from neuralgia, which follow the various ramifications of a nerve, and are of a continuous character, are not generally influenced by so doing. Pains in the eye-ball have not been relieved by this process, except in one or two instances. In cases of periodic neuralgic pains of the head, relief is frequently experienced by carotid pressure, during the attack ; but the same process has no effect in relieving the extreme tenderness of the skin, which often remains during the intervals. In a recent case of a

* I have also made similar experiments on the pulsations of the collaterals at the fingers. At the hand, the ulnar artery supplies the superficial palmar arch and seven internal collaterals; the radial artery supplies the other three collaterals, and the deep arch, which anastomoses freely with the other. Compression of the radial arrested all pulsation of the three external collaterals, and weakened that of the other seven, while compression of the ulnar stopped the seven internal collaterals, and enfeebled the other three. In some cases, however, all the collaterals continued to beat feebly, while the supply artery was compressed.

† Compression of the carotid close under the inferior maxillary, generally excites an involuntary movement of deglutition every time it is applied, which is produced by the irritation of the pharynx, whose fibres are stimulated to act by external pressure, in the same way as when they are excited by the pressure of food. This fact may be usefully applied in case of children or of patients in a comatose state, where there is a difficulty in the administration of food or medicines, for after the liquid has been conveyed to the posterior part of the mouth the application of external pressure on the throat will immediately excite the required act of deglutition.

gentleman who suffered from severe pains over the right eyebrow, every morning at the same hour, I found that carotid pressure immediately relieved him, although when pressure was applied over the part affected, it was found to be exceedingly sensitive.

*Internal pains of the head.*—Dr. Parry states that "headache, whether affecting the external or internal part of the head, is owing to the corresponding conditions of circulation in the internal or external carotid artery. That which occurs from dyspepsia is of the first kind, and may be generally relieved by strong pressure on the carotid arteries, in consequence of which the peristaltic movement of the alimentary canal is often increased, the heat of the head diminished, and the feet, if previously cold, become more warm." (Elements of Pathology.) The sick headache, described by Dr. Fothergill, he specifies particularly as arising from determination of blood to the internal carotid; of the same nature he considers common headache, which affects nervous patients, without sickness. This pain, whether affecting the internal or external parts of the head, he finds to be usually accompanied with strong circulation in the carotids, heat of the head, &c., and to be relieved by cold to the head, a tight bandage, and by compressing the carotids." He observes, (section 718,) that "the term vertigo is applied to two different species of sensation in the head. The first is characterized by a feeling of quick rotation in the inside of the head." It is this species he believes to precede epileptic, paralytic, and apoplectic attacks. "In a moment after its commencement, the patient is seized with nausea, and sometimes with vomiting, and at other times almost immediately falls senseless." I will now mention some cases in which I have tried this process.

Case 1.—Jean Baptiste M——, aged twenty, had been tormented, for many months, with chronic pains in the head, generally on the left side. There had existed for many months on that side a slight discharge from the ear, and in which, on one or two occasions, he had detected small fragments of bone, which appeared to belong to the ossicula auditûs. Hearing was almost entirely lost. These symptoms remained stationary for some months, when suddenly he perceived, one morning, that he had almost lost the use of the right arm, accompanied by a sense of numbness over the skin, and frequent twitching of the muscles of the whole of the right side. After remaining for several days in this condition, he recovered the use of the arm, under the influence of an active treatment. The pains of the head, which I mentioned as being very severe before this attack, lasted for several months after. Generally they were very slight in the early part of the day, but towards the evening they usually became more acute and throbbing. At all times they were liable to be induced by any excitement, after a copious meal, or by an empty stomach. This was among the first cases in which I tried compression of the carotids. The effects of it were (particularly when applied to the side painful) almost entire cessation of the pain, which remission continued for several hours after the pressure was removed. After frequent use of this remedy, he found that the after effect became shorter, and that the cessation from pain only contained so long as the pressure was applied. Ultimately, after the lapse of a year, this patient recovered, with the exception of the loss of hearing on one side.

Case 2.—A gentleman, aged twenty, had severe pains on the left side of the head, deeply seated, and easily induced by mental exertion. He had slight twitchings in the muscles over all parts of the body, and shortly after imperfect hemiplegia and loss of sensibility in the left half of the body. Compression of the carotid, on the same side, invariably stopped the pain, which returned again as soon as the pressure was removed. The twitchings of the muscles appeared so irregularly, that it was impossible to ascertain positively whether it was at all affected by carotid pressure, but it appeared to me not to be influenced by it. I had occasion frequently to examine this patient, and perceived that the pulsations of the carotid were considerably weaker on the side affected than on the other. The radial and collaterals of the fingers on the same side were likewise weaker than on the other.

Case 3.—Mrs. N—— belongs to a family several members of which are very liable to deeply-seated pains occupying one-half of the head. Sometimes half the body, on the corresponding side, has likewise been in pain, accompanied with vertical hemiopia. Mrs. N——, the subject of this case, has experienced all the symptoms above mentioned,—namely, hemiopia, hemicrania, and diminished sensibility on one side of the body. On a recent occasion, she had scarcely recovered from a severe attack of pleurisy, when the head became very painful, the memory much affected, together with most of the symptoms above mentioned, accompanied with vomiting. After all these had been relieved, there remained a feeling of numbness and pricking at the inner surface of the cheek, and part of the throat and tongue; externally, on the same side, one cheek was less sensitive than the other. Carotid pressure stopped the pain in the head, which returned immediately after the pressure was removed.

The three preceding cases present a close analogy to each other. In all, there was pain in the head, generally on one side, deeply seated, and although quiescent during considerable intervals, liable to be called into existence by various causes; and very probably, if the internal parts could have been examined, we should have found a portion of the membranes, and of the cerebral substance itself, congested with blood. In case 1, the internal ear appears to have been the main cause of the congestion. Carotid pressure in all three caused a cessation of the pain; with this difference, however, that in the first the effect was much more permanent than in the others. In case 2, the carotid was weaker on the side affected. This is a point which deserves great attention, but at the same time is surrounded with considerable difficulties. Dr. Parry appears to have met with frequent cases of this sort. He mentions, in particular, that of a gentleman suffering pain on one side of the head, and in the eye of the same side. On the opposite side of the body, there was hemiplegia, and the carotid was preternaturally strong. But unless the case is as decided as this, or as case 2, there is much difficulty in ascertaining the comparative strength of the carotid. Careful injection of these arteries after death might be a means of determining this point positively. The effect of a loaded stomach, in inducing congestion of the brain in apoplexy, has not escaped popular observation; but the contrary condition of that organ (the stomach) is not less effectual in causing pain in certain congestive conditions of the brain. The cerebral symptoms which occur in starvation are well known.

The following case is interesting, as it shows that compression of the carotids does not always alleviate pain arising from congestion of the brain :—

Case 4.—Mrs. H—— had been suffering from severe pain over the forehead, spreading sometimes over the entire head, which was aggravated by the slightest movement of the head. At the same time, there existed giddiness and loss of memory. Carotid pressure always appeared to increase the pain. She informed me that she had omitted to be bled, as had been her custom once a year, and the catamenia were absent. The pain in the head was relieved by abstraction of blood from the arm. On the following day, after a violent attack of hysteria, inflammation of the brain declared itself, accompanied with hemiplegia; and imperfect anæsthesia of the right side. After a prolonged illness, during which an antiphlogistic treatment was had recourse to, she recovered the use of her limbs. There still remained a severe pain in the head, which carotid pressure had the effect of relieving.

Case 5.—Margaret ——, aged nineteen, strong, and of a plethoric habit, had a severe attack of indigestion after eating large quantities of fruit. After rejecting the irritating substance from the stomach, there remained constant nausea, and repeated efforts at vomiting, which continued upwards of two hours, after the stomach had been emptied, notwithstanding the internal and external means resorted to. On account of some violent pains in the head, I was led to try compression of the carotid, which immediately relieved the pain, but also, to my surprise, the vomiting ceased. On removing the pressure, the pain and sickness returned immediately. The same effects were produced by removing and applying pressure several times. I found it desirable to remove blood from the arm, which afforded immediate relief to the pain and vomiting.

This case is the more remarkable, as I have not generally found that vomiting is relieved by carotid pressure. It is well known that venesection, when carried to a certain extent, frequently causes nausea and vomiting ; and when the bleeding is carried on still further, syncope and convulsions will ensue. Again, patients suffering from violent and habitual pains of the head, when there exists no general excess of blood in the system, not uncommonly complain of considerable nausea when the carotids are compressed. In the case I am now about to relate, carotid pressure acted in such a manner as might have been expected from a considerable loss of blood.

Case 6.—Mr. W————, a person of drunken habits, complained of constant heaviness and deeply seated pains in the head, increased by stooping and by compression on one of the carotids. About six ounces of blood were taken from him, while he was in a standing position, which caused him to become very weak, and the vein was closed. In the course of half an hour he recovered, and was able to walk. Compression being applied at this time on one of the carotids, caused immediate syncope. On removing the compression he speedily recovered. A second application was attended with the like results, only in a less violent degree. On a third occasion it appeared to have no effect whatever.

In cases of hysteria and epilepsy compression of the carotids frequently exerts a most remarkable influence, when performed during the attack, in shortening the duration or in diminishing the violence of the convulsions.

Case 7.—Miss M—— has suffered from attacks of hysteria for two years, accompanied by strong convulsions and slight foaming at the mouth. I had recourse to pressure on the carotids; and after it had been applied a few moments, the patient began to look about and recognise those around her. By continuing it she recovered entirely, though remaining much exhausted. Within the space of a few hours after she had two other slighter attacks, which were easily relieved in the same manner. As I have before said, this process is very beneficial in epileptic fits; but the contraction of the muscles of the whole frame, and especially of those belonging to the neck, frequently renders it impossible to continue it for a sufficient length of time.

Case 8.—William F——, aged 30, four years ago had brain fever, followed by paralysis of the left side, from which he completely recovered. A few months after this he had his first epileptic attack. The premonitory symptoms were, generally, heaviness and pains in the head, alleviated by pressure on one of the carotids, and slight twitchings over various parts of the body. At the time of the convulsions, compression on one of the carotids calmed the violence of the spasms, and he soon settled into a state of lassitude and heaviness, which shortly terminated in a troubled sleep. Dr. Parry relates, that in a severe case of catalepsy, accompanied with total insensibility, pressure on both carotids uniformly suspended the symptoms and restored the patient's senses, and that pressure on one carotid had no perceptible effect, that a moderate degree of pressure on the carotids removed the catalepsy, and induced convulsions, while a greater degree removed the convulsions also. The converse order of occurrences was equally true.

Case 9.—Mr. P., in consequence of an attack of congestion to the brain, had the right side nearly paralyzed, and at intervals the right arm was agitated by a tremour which he was unable to control; at the same time, there existed violent pain on the right side of the head, and the power of hearing on the same side was nearly lost. Carotid pressure on the side affected gave relief, and on the other side still greater, and caused the shaking of the arm to cease for a few minutes each time it was performed. A few ounces of blood taken from the arm until faintness ensued diminished the pains, and freed him from the spasmodic trembling in the arm. This last symptom appeared again after the lapse of two hours, and, after continuing about an hour, suddenly ceased, and did not return. A son of Mr. P. was affected on two occasions with epileptic fits, which had been preceded by violent pains in the feet. The fits were not relieved by carotid pressure, but were stopped on each occasion by tight ligatures round the legs.

As a remedial agent, compression of the carotids cannot, in its present condition, be ranked with others which are intended to effect a permanent cure of disease. Its application is limited to those cases, not unfrequently to be met with in practice, when, other agents having been found powerless, this process provides us with an active means of shortening and dispelling a series of formidable symptoms distressing to witness. Under this point of view its sphere of usefulness, though bounded, is important, inasmuch as it is a matter of experience that hysteria, epilepsy, and spasmodic affections in general, are more likely to disappear altogether in proportion as their attacks are rendered less frequent, and as they have less obtained a "droit de domicile" in the economy.

For diagnosis it gives us several indications of importance. By restricting our attention to its effects on pains of the head of every description, we may distinguish three kinds of headache, according as they are either removed, remain unaffected, or are increased, by arresting the current of blood within the carotids. The pains of the first kind are generally of a throbbing nature, increasing with the systole of the heart. Those of the second appear to arise from some local action in the tissue affected, which is independent of vascular congestion. These pains are generally much more diffused over the head than the former, they continue longer, and are less likely to exacerbate at intervals. The third kind includes most of those pains which occur from anæmia, or from the blood not possessing some of its usual elements, as in chlorosis. It comprises, likewise, certain congestive states of the brain, where pressure does not remove but increases the pain. In the foregoing cases, I have mentioned some instances of this sort which I have found to be benefited by bleeding. It is impossible at present to explain satisfactorily these exceptions, which are not unfrequent, but I have often found them combined with general plethora. With respect to the spasmodic diseases, where compression sometimes acts in a way so immediate and efficient, and at other times not at all, a more full investigation will very probably enable us to find the part of the nervous system primarily affected.

Considered as a physiological experiment, carotid compression presents an unlooked-for means of testing some opinions concerning the action of the brain, and of the cerebral hemispheres in particular. Aristotle and Galen were already aware that artificial irritation of the brain caused no pain, and modern physiologists, in confirming their statements, have ascertained that neither mechanical, chemical, nor other modes of irritation of the cerebral hemispheres and cerebellum ever cause convulsive movements or pain. On the other hand, they have discovered that irritation of the spinal marrow, or of the medulla oblongata, invariably causes convulsive action. The discoveries of Dr. Marshall Hall have shown, physiologically, the line of demarcation existing between these two divisions of the nervous system. He has demonstrated that the spinal marrow, far different from being merely a large nerve, containing in one sheath all the nerve tubules ascending to the brain, possesses distinct faculties, which enable it to act as a medium of communication between the separate nerve tubules which it receives from all parts of the system. For the physiologist and the pathologist it is impossible to overrate the importance of the reflex actions and the excito-motory systems. It has served to guide us in the intricate course of the various internal impressions, from the points at which they take their origin until their final termination within the muscular fibre. But can we go to the extent of affirming, that in all cases of epilepsy, convulsions, hysteria, &c., are the results of a reflex action of the spinal marrow, or of a direct irritation upon it. I think it impossible, in face of the preceding observations, to deny to the cerebral hemispheres the power, in certain morbid conditions, of also giving rise to similar increased muscular action.

The negative results of artificial irritation of the brain in animals cannot be considered as invalidating the positive results of these experiments made on our own species. In drawing conclusions with respect to the functions of the brain, from experiments, it must not be forgotten that the irritation we are able to excite is far different from that arising from a morbid condition. In vivisections the irritation is of that violent nature, that the part experimented upon may be considered as "struck with death," and no longer capable of continuing its functions in any way. In disease, on the contrary, the excitement which causes epilepsy and other convulsive conditions, appears to be general and diffused either in one or both hemispheres, and sufficient only to exalt the natural functions. The effect is, to cause increased manifestations of the mental faculties, arousing the images treasured in the cerebrum. When carried a degree farther, the excitement of the cerebral tissue, no longer contained in these bounds, becomes capable of being transmitted downwards to the rest of the encephalon.

In confirmation of these ideas, how frequently we find each return of the catamenia attended with mental excitement and perversion of the moral sentiments, terminating often in a violent attack of hysteria. The heat and injection of the countenance, the suffusion of the conjunctiva, surfaces supplied by branches from the same arterial trunk, furnish us with correct indications of the state of the parts within the cranium. It is in this condition, by restoring the equilibrium in the various parts of the vascular system, by moderating the impetus of the blood in the branches of the carotid, that we are enabled, as if by a charm, to subdue the whole train of morbid symptoms which have arisen.

1, St. Mary Abbot's terrace, Kensington,
August, 1848.

